# Bargaining risk governance within the context of China’s belt and road initiative: Perspectives derived from the Kindleberger Trap theory

**DOI:** 10.1371/journal.pone.0332379

**Published:** 2025-09-25

**Authors:** Zhifeng Shen, Yongming Ye, Ahsan Siraj, Zhihui Li

**Affiliations:** School of management, Zhengzhou University, Zhengzhou, Henan, China; Nanjing Audit University, CHINA

## Abstract

“Belt and Road” infrastructure projects frequently encounter bargaining risks. This study aims to explore the causes of bargaining behavior and investigate how to control bargaining behavior in projects. The study identifies the factors affecting bargaining risk and employs evolutionary game theory to examine the strategic decisions of Chinese contractors and host governments. The results show that lower negotiation and bargaining costs promote sustained cooperation in the negotiation process. When the value of the contract is substantial and the contractor receives less benefit from the contract than it claims from the host government, terminating the project is good for the contractor.

## 1. Introduction

Mega projects are often empirically defined as a complex system with budgets over US$1 billion. Mega projects are implemented according to design documents, unified economic accounting, independent administrative organization and unified management of the project, with large investment scale, high technical requirements, compact and complex structure, difficult management, wide professional knowledge, many participating units, complex construction environment [1,2]. The construction of mega projects is carried out under the conditions of certain human, material and financial resources to achieve the investment goal. Most of the “Belt and Road” infrastructure projects are mega projects.

The infrastructure initiatives known as the “Belt and Road” have yielded significant outcomes, including in the export of global public goods [3]. However, as of 2021, 234 undertakings have encountered the potential for bargaining risks. As a result of the influence of certain foreign scholars and media discourse, the ruling party of the host country, opposition parties, local government, opinion leaders, and residents have initiated a bargaining approach, citing poor project returns, loss of sovereignty, national debt crisis, state-owned enterprise monopolies, environmental concerns, lack of transparency, and subpar quality of Chinese infrastructure construction [4,5]. Kindleberger, a top expert on international issues and a major constructor of the Marshall Plan, pointed out that moments of the rise of emerging powers and the decline of established powers often lead to a lack of supply of global public goods. In 2017, the American political scientist Joseph Nye formally put forward the Kindleberger Trap, arguing that changes in the power of China and the United States and the continuation of the game have resulted in the United States inability to assume responsibility for the supply of global public goods and that for a variety of reasons China is unable or unwilling to supply such public goods. This imbalance in responsibility and legitimacy creates an institutional vacuum, which in turn allows weaker actors—such as host country governments, local authorities, and non-state stakeholders—to strategically initiate bargaining behavior. In the context of the Belt and Road, this manifests in the re-negotiation of previously agreed terms such as pricing, governance structures, or environmental standards. From this perspective, the bargaining risk observed in BRI (the “Belt and Road” Initiative) infrastructure projects can be seen as a micro-level manifestation of the Kindleberger Trap: the structural tension between rising responsibility and insufficient authority leads to governance fragility and contract instability. The above theory explains the dilemma faced by the “Belt and Road” infrastructure projects. The bargaining entities often alter previously determined contract terms, such as the price or value of the project, in violation of local laws, regulations, and international contracting practices. In recent years, there has been a significant increase in the number of infrastructure projects with bargaining behavior in regions such as Malaysia, Myanmar, Thailand, Sri Lanka, and others along the “Belt and Road” [6–9].

Unlike Western management concepts and theories that rely on strict adherence to contractual obligations and relevant FIDIC clauses to resolve disputes, bargaining behavior is a departure from these traditional provisions. These concepts and standards have limited applicability in controlling the bargaining behavior of the “Belt and Road” infrastructure projects.

To address this issue, it becomes imperative to introduce a new governance framework that not only identifies bargaining as a distinct form of project risk but also accounts for the structural and relational dynamics that drive it.

The Nobel Prize in Economics was awarded to two scholars in auction theory for their theoretical and practical innovations in bargaining in 2020 [10]. There exists no research that provides a conceptual elucidation of the bargaining behavior in the infrastructure projects of the “Belt and Road”, but solely describes it through case studies [11,12].

Therefore, this study proposes to conceptualize and model bargaining risk as an independent and quantifiable governance challenge, and to explore its dynamics through a combination of case-based evidence, evolutionary game theory, and Eastern governance frameworks. This effort is of both theoretical and practical value, as it contributes to the global understanding of how infrastructure diplomacy unfolds in the absence of hegemonic governance certainty.

## 2. Relevant theories and literature review

### 2.1 The bargaining behavior of the “Belt and Road” infrastructure projects

The article by Glosten and Milgrom marked the inception of highly cited literature in the realm of bargaining research [13]. Since then, the subject matter of bargaining behavior has been extensively explored, as evident from the works of Carr [14], Cui and Wiggins [15], Khezr and Menezes [16], Lester, Visschers, and Wolthoff [17], and Khezr and Menezes [18]. The research has encompassed diverse aspects of bargaining, ranging from its form to its effect, and has involved the negotiation of general consumer goods, financial products, real estate, and service products. Furthermore, the theories of auction [Auction theory: Auction theory is a theoretical tool used to guide the design of real-world auctions that can help sellers increase their income while allowing buyers to buy at a lower cost, and the seminar between buyers and sellers about price is an economic equilibrium.] and bargaining have found relevance in the context of bidding for infrastructure projects and negotiating engineering projects [19]. These theories also provide an explanation for the behavior of the “five parties” [Five parties: five parties responsible for the construction of the main project are to undertake the construction of construction projects: the construction unit project leader, survey unit project leader, design unit project leader, construction unit project manager and supervision unit superintendent engineer.] involved in infrastructure projects. However, the “Belt and Road” doctrine and public opinion may stimulate the bargaining behavior in these projects, which is primarily linked to regime change, debt crisis, employment deterioration, environmental crisis, and misleading media propaganda in the countries along the “Belt and Road”. To varying degrees, the “Belt and Road” has enhanced the bargaining power and development opportunities of many countries in the global South [20]. However, the deliberate bargaining behaviors often portray Chinese general contracting enterprises as threats from Chinese state-owned enterprises, characterized by low-quality standards, corruption in projects, plunder of resources, sovereignty crisis, lease-term colonization, and pan-politicization. These behaviors may trigger bargaining behaviors against Chinese state-owned enterprises by local governments, other organizations, or local residents at any time. Bunnak and Song analyze governance and renegotiation dynamics in the Sino–Thai railway project, highlighting how shifting stakeholder roles and institutional adaptation influence contract modification behaviors [21]. Similarly, Wang investigates Southeast Asian countries’ policy responses to contractual uncertainties, revealing strategies such as proactive coordination and procedural flexibility—practices that closely resemble the governance challenges associated with bargaining behaviors in infrastructure projects [22]. These recent empirical insights offer valuable perspectives for expanding the conceptual scope of BRI-related risk governance, nevertheless, no scholars have systematically studied this behavior, its causes, and its hazards.

### 2.2 The bargaining risk of the “Belt and Road” infrastructure projects

The potential for the bargaining risk within the “Belt and Road” infrastructure initiatives arises from the bargaining behavior of the host government. This behavior is demonstrated through the inconsistent stance of the host government towards the “Belt and Road” program. The host government’s contradictory attitude of seeking to collaborate with Chinese businesses to enhance its infrastructure whilst also suspending or terminating infrastructure initiatives that are already underway for certain reasons, is rooted in the current global political landscape [23]. The year 2023 will mark the 10th anniversary of the “Belt and Road” initiative, and China has entered into over 200 cooperation agreements with 151 countries and 32 international organizations for the construction of the “Belt and Road”. During the process of state interaction, smaller and medium-sized countries with significant autonomy will utilize negotiation tactics toward larger nations in the pursuit of their own interests. For, example, Kazakhstan’s multi-vector State-to-State engagement with other countries, such as China, has recently become an important strategy for enhancing its bargaining power. China’s willingness to connect in the “Belt and Road” also gives the Kazakh government more bargaining leverage [24]. This represents a bargaining tool of the host government and can be interpreted as a “breach of contract at the negotiating table”. Although the “Belt and Road” infrastructure projects are paused, most of them are resumed through negotiation, to some extent complying with certain bargaining terms. Nevertheless, the bargaining behavior not only delays the completion date of the infrastructure projects and increases the overall contracting cost for the Chinese party, but also harms the international reputation of the bargaining party. Based on the above analysis of the bargaining behavior, this research defines the notion of bargaining risk within the “Belt and Road” infrastructure initiatives as the demand for a modification of the project price or value initiated by bargaining party to the Chinese general contractor subsequent to the signing of the construction contract of the “Belt and Road” infrastructure projects. This poses a significant risk to the prospects of the infrastructure project.

### 2.3 Causes of the bargaining risk of the “Belt and Road” infrastructure projects

The variability in cost or value of infrastructure projects in the “Belt and Road” initiative shows a strong correlation with political risk, as posited by Robock [25]. Although “bargaining risk” overlaps with “political risk” in the traditional sense in some ways, there are obvious differences between the two in terms of their essential characteristics and impact mechanisms. Bennon and Fukuyama [5] conducted an in-depth analysis of the “obsolescing bargain” mechanism in Belt and Road Initiative projects, pointing out that when initial cooperative arrangements become ineffective under a new government or regime, Chinese enterprises often face a new round of “re-negotiation” dilemmas. Although their study highlights the renegotiations triggered primarily by political regime changes—characterized as abrupt, coercive, and externally imposed—the “bargaining risk” defined in our research differs distinctly. Specifically, Political risk refers to the uncertainty that political events or government actions pose to production and operations, and national strategies drive subject behavior., which usually stems from changes in government, legal changes, or sudden policy interventions, and is characterized by its non-institutional, unpredictable, and coercive nature [26]. For instance, Southeast Asian nations have persistently sought alterations in the price or value of the “Belt and Road” infrastructure projects, which are linked to the change of local regimes or ruling parties, as indicated by Li et al. [27]. Bargaining risk, on the other hand, is a negotiating tactic, and interest motivates the subject behavior. mainly manifests itself in the host country’s proactive bargaining demands based on project value reassessment or political and economic motives during project implementation. It has a certain degree of predictability and strategic game-playing nature, and is often embedded in institutional frameworks and contractual relationships.

Notably, the involvement of the Chinese government has led to a decline in such risks, so effective risk management of such projects is likely to bring significant benefits to Chinese enterprises, as argued by Conway [28]. Moreover, smaller countries also aim to change the contract price or value terms to achieve more benefits, such as resolving industrial structure issues, introducing advanced technologies, tackling employment difficulties, and enhancing their ecological environment through the construction of “Belt and Road” infrastructure projects. Using a strategic action area framework to analyze Chinese investors’ interactions with Pakistan’s power sector, Safdar M T confirms the turnover of Chinese investors’ bargaining power at different stages of a project, which tends to be greater at the beginning of a project’s investment and is often reversed afterwards [29]. Consequently, the factors that influence bargaining behaviors can be highly intricate. Existing literature mostly considers bargaining behaviors as political risks, they often overlook the distinction between the two. Therefore, this research seeks to identify the crucial factors that give rise to bargaining risks associated with the “Belt and Road” infrastructure projects, commencing with a thorough examination of the manifestation of bargaining behavior.

### 2.4 Control of the bargaining risk of “the Belt and Road” infrastructure projects

There exists a dearth of literature pertaining to the management of bargaining risk associated with “the Belt and Road” infrastructure projects, with the few available studies being primarily focused on project management, thus causing confusion. Existing research fails to adopt a macroscopic and project perspective to manage different bargaining behaviors and conceptualize the bargaining behavior. The current dispute resolution mechanism in the international engineering field or in the larger international trade field relies on Western concepts which are inefficient in controlling the bargaining behavior of the “Belt and Road” infrastructure projects.

Through the review of the relevant literature, it can be found that the resolution of the bargaining risk of the “Belt and Road” infrastructure projects generally relies on international commercial rules and international contract terms. However, the host countries of the “Belt and Road” infrastructure projects are generally developing countries with undeveloped market economy, and international business rules and international contract terms are not fully applicable in solving the bargaining risk of the “Belt and Road” infrastructure projects [30]. Therefore, under the premise of international commercial rules and international contract terms, the Eastern governance theory can be introduced to solve the bargaining risk of the “Belt and Road” infrastructure projects, which can improve the solution efficiency and reduce the cost, and supplement the governance theory of international infrastructure projects.

## 3. Case analysis of the bargaining risk of the “Belt and Road” infrastructure projects

The analysis was carried out in accordance with the exemplification of “Belt and Road” infrastructure bargaining projects, involving the refinement of bargaining categories, purification of bargaining factors. This culminated in the creation of a comprehensive categorization table, which is meticulously detailed in Table 1.

**Table 1:** Case analysis of the bargaining risk.

No.	Project names	The “Belt and Road” infrastructure bargaining project examples extraction	Bargaining categories refinement	Bargaining factors purification
1	Colombo Port City Project in Sri Lanka	Incoming President Sirisena’s viewed that the Colombo project was started during the former president’s administration and was suspected of violating local laws and circumventing relevant environmental requirements; Sri Lanka’s excessive borrowing from China and the need to reconsider the Colombo Port City project.	Review of the license certificate;Unknown contract terms; Debt relief.	Political factor; Economic factor;Legal factor.
2	Malaysia East Coast Railway Plan	During the general election campaign, Mahathir opposed the foreign policy of the then ruling party, arguing that corruption was involved and detrimental to Malaysia’s development, and called for a scaling down of the project; Najib set up companies for money laundering and corruption, and the East Coast Railway project was questioned and opposed; Chinese-funded projects led to Malaysia’s debt problems.	Reduced project scale; Project cost reduction; Debt trap.	Political factor; Economic factor; Legal factor.
3	Jakarta-Bandung High-Speed Railway in Indonesia	The “grassroots” President Joko’s use of the project as a political chip for re-election caused discontent among the elite, and “anti-Joko” forces used the media and academics to smear the Jakarta-Bandung High-Speed Railway project; Unclear land ownership increases the cost and difficulty of project land acquisition; With rising cost, China-Indonesian joint ventures are facing huge capital pressure and financing risks.	Certificates are not yet complete; Project scale down; People’s livelihood compensation issues; Insufficient funds.	Political factor; Economic factor; Cultural factor.
4	China-Thailand Railway	Due to the coup in Thailand, the strategic project of China-Thailand Railway has no time to be taken into consideration; Thailand’s financial system is unstable, and there are problems with railway financing and cost recovery; China’s interest rate on loans for the China-Thailand railway is higher than other countries’; Construction and maintenance are difficult, and the risk of land expropriation is high.	High interest rates on Chinese loans; Project scale down; Lack of detailed assessment.	Political factor; Economic factor; Cultural factor.
5	Myanmar Myitsone Hydropower Station	The Myanmar party believes that the construction of the project has deteriorated the living environment of fish and endangered the water quality downstream; The hydropower station is located on an earthquake fault, which creates a major potential hazard to people’s lives; the Myitsone region is the birthplace of local civilization and the local people oppose the construction of the hydropower station; The Myitsone project has become the main topic of political games between the local government, armed forces and ethnic forces.	Environmental compensation issues; Cultural difference issues; Internal benefit distribution issues.	Environmental factor; Cultural factor; Political factor.
6	China-Myanmar Kyaukpyu-Kunming Railway	Myanmar citizens and NGOs have repeatedly protested to the Myanmar government over environmental protection, land compensation, employment and other issues, demanding the suspension of the railroad project; Myanmar’s media smear China as “interfering in Myanmar’s internal affairs and threatening Myanmar’s national security”; Chinese enterprises have left the negative impact of not paying attention to the environment and disregarding people’s livelihoods.	People’s livelihood compensation issues; Differences in technical standards; Environmental pollution problems.	Legal factor; Environmental factor; Political factor.

(Source: The paper draws up this table by analyzing the literature, news, books and other materials)

The bargaining behavior of the “Belt and Road” infrastructure projects is a renegotiation process, the process of the bargaining will involve the two sides of the dynamic game, so the evolutionary game theory can be applied to analyze the game process of the two sides.

## 4. Method

This paper uses an evolutionary game method to analyze the bargaining strategies of the host government and Chinese engineering contractors in the “Belt and Road” infrastructure projects.

### 4.1 Basic hypotheses

Hypothesis 1 posits that, during negotiations for “Belt and Road” infrastructure projects, the host government’s strategy space can be defined as either “continued cooperation” or “terminated cooperation”, abbreviated as “{$A_{1},\;A_{2}$}”. The former denotes a willingness to continue the project while initiating bargaining behavior with the Chinese general contractor, while the latter signifies a refusal to continue construction and payment of compensation to said contractor. Similarly, the Chinese general contractor’s strategy space can be categorized as either “continued cooperation” or “terminated cooperation”, abbreviated as “{$B_{1},\;B_{2}$}”. The former denotes a desire to continue the project and accept negotiations initiated by the host government, while the latter signifies a refusal to continue construction and initiation of a claim against the host government.

Hypothesis 2 assumes that, if the host government expects to complete the project, the loss it will suffer can be quantified as “{q(q>0)}”. This loss can be reduced by negotiating a difference in contract price with the Chinese general contractor, denoted as “{p(p>0)}”. If negotiations break down and the project is canceled, the host government can obtain savings of “{q}”, which is based on the assumption that there is “{p>q}”.

Hypothesis 3 establishes that the total benefit to both the host government and the Chinese general contractor, in the event of successful cooperation, is “{$\pi_{g}$}” and “{$\pi_{e}$}”, respectively.

Hypothesis 4 suggests that the cost of performance for either the host government or the Chinese general contractor, due to a desire for continued cooperation, is “{$c_{g},c_{e}$}”. This includes the human and material resources expended by both parties during negotiations.

Hypothesis 5 claims that, among the host government and the Chinese general contractor, the party that chooses terminated cooperation will suffer a loss of credibility, quantified as “{μ,θ}”.

Hypothesis 6 states that, if negotiations between the two parties break down, the host government will compensate the Chinese general contractor in the amount of “{d}”.

Hypothesis 7 indicates that, if negotiations between the two parties break down, the Chinese general contractor will lose potential profits in the host market, which is estimated to be “{l}”.

Based on these hypotheses, the payment matrix for the evolutionary game of bargaining negotiation is constructed, as detailed in Table 2.

**Table 2:** Definition of key variables in evolutionary game model.

Symbol	Definition
q	The loss when the host government completes the project
p	The host government reduces losses by negotiating the differences in contract prices with the Chinese general contractor
$\pi_{g}$	The total benifit of the host government when successfully cooperating with the Chinese general contractor
$\pi_{e}$	The total benifit of the Chinese general contractor when successfully cooperating with the host government
$c_{g}$	The bargaining cost of the host government, includes the human and material resources expended during negotiations.
$c_{e}$	The bargaining cost of the Chinese general contractor, includes the human and material resources expended during negotiations.
μ	The loss of credibility of the host government
θ	The loss of credibility of the Chinese general contractor
d	The amount compensated by the host government to the Chinese general contractor when the negotiations between the two parties broke down
l	The potential host market loss of Chinese contractors

The evolutionary game payment matrix of the bargaining negotiation between the host government and Chinese general contractor is shown in Table 3.

**Table 3:** Bargaining negotiation evolutionary game payment matrix.

Both parties of the game		Chinese general contractor	
**continued cooperation**	**terminated cooperation**
**Host Government**	**continued cooperation**	$\begin{array}{c} \pi_{g}+p-\mu -c_{g}\\ \pi_{e}-p+\theta +l-c_{e} \end{array}$	$\begin{array}{c} -c_{g}-\mu +q-d\\ -\theta +d-l \end{array}$
	**terminated cooperation**	$\begin{array}{c} -\mu +q-d\\ -c_{e}+\theta +d-l \end{array}$	$\begin{array}{c} -\mu +q-d\\ -\theta +d-l \end{array}$

### 4.2 Solving for the equilibrium point of bargaining negotiation

Assuming that the probability of the host government adopting the continued cooperation strategy in the initial state is x(x∈[0,1]) and the probability of adopting the terminated cooperation strategy is 1−x. Based on the payment matrix, we can obtain the expected benefits $E\left(A_{1}\right),E(A_{2})$ and the average benefits E(A) of the host government adopting the continued cooperation and terminated cooperation strategies, respectively:


$$E(A_{1})=y(\pi_{g}+p-\mu -c_{g})+(1-y)\left(-c_{g}-\mu -d+q\right)$$ (1)

$$E\left(A_{2}\right)=y(-\mu -d+q)+(1-y)(-\mu -d+q)$$ (2)

$$E(A)=xE(A_{1})+(1-x)E(A_{2})$$ (3)

The replication dynamic equation for the host government’s strategy at this point is:


$$F(x)=dx/dt=x\left[E(A_{1})-E(A)\right]=x(1-x)\left[-c_{g}+(\pi_{g}+p+d-q)y\right]$$ (4)

Assuming that the probability of the Chinese general contractor adopting the continued cooperation strategy in the initial state is y(y∈[0,1]) and the probability of adopting the strategy is 1−y. Based on the payment matrix, we can obtain the expected benefits $E(B_{1}),E(B_{2})$ and the average benefits E(B) for the Chinese general contractor adopting the continued cooperation and terminated cooperation strategies, respectively.


$$E(B_{1})=x(\pi_{e}-p+\theta +l-c_{e})+(1-x)\left(\begin{array}{c} -c_{e}+\theta +d-l \end{array}\right)$$ (5)

$$E(B_{2})=x\left(\begin{array}{c} -\theta +d-l \end{array}\right)+\left(\begin{array}{c} 1-x \end{array}\right)\left(\begin{array}{c} -\theta +d-l \end{array}\right)$$ (6)

$$E(B)=yE(B_{1})+(1-y)E(B_{2})$$ (7)

The replication dynamic equation of the Chinese general contractor’s strategy at this point is:


$$F(y)=dy/dt=y[E(B_{1})-E(B)]=y(1-y)\left[2\theta -c_{e}+(\pi_{e}-p-d+2l)x\right]$$ (8)

When the above dynamic equation F(x)=0,F(y)=0, it means that the infrastructure project offer negotiation evolution system reaches a relatively stable state, and the five local equilibrium points are solved as (0,0), (0,1), (1,0), (1,1), (1,1), $\left(x^{*},y^{*}\right)$, where $x^{*}=\frac{c_{e}-2\theta }{\pi_{e}-p-d+2l},y^{*}=\frac{c_{g}}{\pi_{g}+p+d-q}$. Only the cases of x,y∈[0,1] are considered.

### 4.3 Equilibrium state analysis of bargaining negotiation

The evolutionary steady state of the replication dynamic equations can be obtained from the local stability analysis of the Jacobian matrix. The Jacobian matrix of the bargaining negotiation evolution system of the “Belt and Road” infrastructure projects is shown in Formula (9).


$$J(x,y)=(\begin{array}{cc} (1-2x)\left[\left(\pi_{g}+p+d-q\right)y-c_{g}\right] & x(1-x)\left(\pi_{g}+p+d-q\right)\\ y(1-y)\left(\pi_{e}-p-d+2l\right) & (1-2y)\left[2\theta -c_{e}+\left(\pi_{e}-p-d+2l\right)x\right] \end{array})$$ (9)

If the Jacobian matrix satisfies det.J>0 and trJ<0, the equilibrium point obtained by the solution of the replication dynamic equation is the bargaining negotiation system evolutionary stability strategy (ESS). The results are calculated by substituting each local equilibrium point into the expressions of the determinant of Jacobian matrix (det.J) and the trace of the Jacobian matrix (trJ). The details are shown in Table 4.

**Table 4:** Determinant and trace of Jacobian matrix.

Equilibum points	det.J	trJ
(0,0)	$c_{g}(c_{e}-2\theta )$	$2\theta -c_{e}-c_{g}$
(0,1)	$\left(\begin{array}{c} \pi_{g}+p+d-q-c_{g} \end{array}\right)\left(\begin{array}{c} c_{e}-2\theta \end{array}\right)$	$\pi_{g}+p+d-q-c_{g}+c_{e}-2\theta$
(1,0)	$c_{g}(\pi_{e}-p-d+2l+2\theta -c_{e})$	$c_{g}+\pi_{e}-p-d+2l+2\theta -c_{e}$
(1,1)	$\left(\begin{array}{c} q+c_{g}-\pi_{g}-p-d \end{array}\right)\left(\begin{array}{c} -\pi_{e}+p+d-2l-2\theta +c_{e} \end{array}\right)$	$q+c_{g}+c_{e}-\pi_{g}-\pi_{e}-2\theta -2l$
$\left(x^{*},y^{*}\right)$	$\frac{c_{g}\left(c_{e}-2\theta \mathrm{\backslash rightleft}(\pi_{e}-p-d+2l+2\theta -c_{e}\right)\left(\pi_{g}+p+d-q-c_{g}\right)}{\left(\pi_{e}-p-d+2l\right)\left(\pi_{g}+p+d-q\right)}$	0

From Table 4, it can be seen that the steady state of the evolving system is determined by the positive and negative cases of $\left(c_{e}-2\theta \right)$, $\left(\begin{array}{c} \pi_{g}+p+d-q-c_{g} \end{array}\right)$ as well as $\left(\pi_{e}-p-d+2l+2\theta -c_{e}\right)$. To facilitate the discussion of the system evolution results below, let $r_{1}=\pi_{g}+p+d-q-c_{g}$ and $r_{2}=\pi_{e}-p-d+2l+2\theta -c_{e}$. Where $r_{1}$ denotes the difference between the benefits of continuation and termination of the contract by the host government, and $r_{2}$ denotes the difference between the benefits of continuation and termination of the contract by the Chinese general contractor. Based on the positive and negative cases of each, five evolutionary scenarios are obtained in combination for discussion.

### Situation 1:$c_{e}-2\theta >0,r_{1}>0,r_{2}>0$

**Fig 1:**
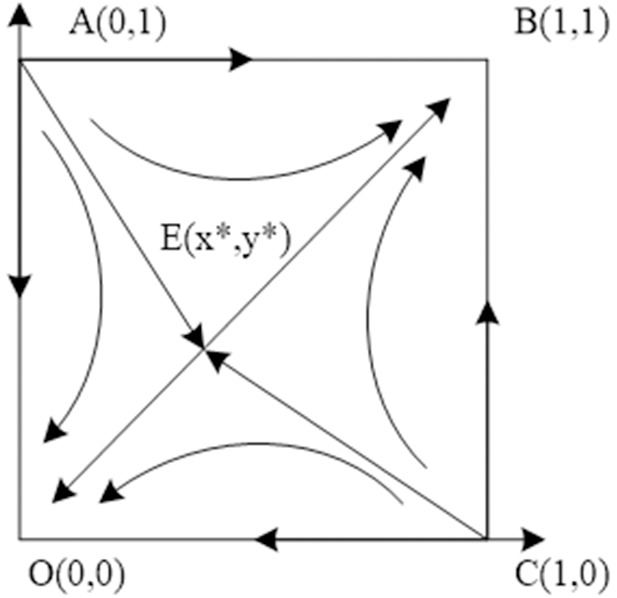
Situation 1 evolutionary phase diagram.

At this time, the performance cost of the Chinese general contractor is higher than 2 times the loss of reputation and the gain of the host government to continue the contract by way of bargaining is greater than the gain of termination, and the gain of the Chinese general contractor to accept the way of bargaining to continue the contract is greater than the gain of compensation from suing the host government. The evolutionary stability of the system is shown in Table 5, and the evolutionary path is shown in Fig 1.

**Table 5:** Stability analysis of equilibrium points of Situation 1 evolutionary game system.

Equilibrium points	det.J	trJ	Stability
(0,0)	+	–	ESS
(0,1)	+	+	Instability point
(1,0)	+	+	Instability point
(1,1)	+	–	ESS
$\left(x^{*},y^{*}\right)$	–	0	Saddle point

Under this condition, both B (1,1) and O (0,0) are the equilibrium points of the system. The line segment formed by connecting the saddle points A, C, E divides the plane into regions 1 and 2. When the initial state is in region 1, the evolutionary stability direction of both parties of the game is O (0,0); when the initial state is in region 2, the evolutionary stability direction of both parties of the game is B (1,1). It can be seen that the equilibrium strategies of the two evolutionary paths are related to the relative area sizes of region 1 and region 2. When the area of region $S_{1}>S_{2}$, the probability of the initial state falling in region 1 increases and the probability of the game equilibrium of the host government and the Chinese general contractor converging to O (0,0) increases; when the area of region $S_{1}<\;S_{2}$, the probability of the initial state falling in region 2 increases and the probability of the game equilibrium of the host government and the Chinese general contractor converging to B (1,1) increases. The analysis shows that when both parties receive more from the project, the greater the potential market profit of the host country and the higher the reputation of the Chinese general contractor, that is, $\pi_{g}$, $\pi_{e}$, l, θ are larger. The smaller the negotiation cost of both parties, that is, $c_{g}$, $c_{e}$ are smaller, the greater the probability that the game equilibrium converges to B (1,1).

### Situation 2:$C_{e}-2\theta >0,r_{1}<0\;or\;r_{2}<0$

**Fig 2:**
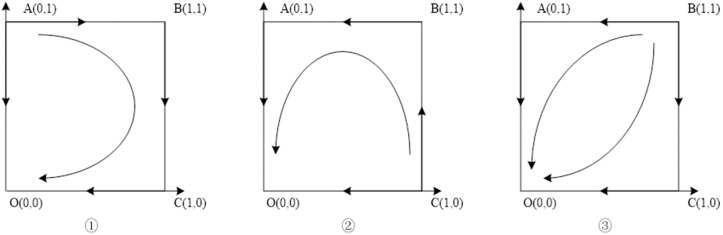
Situation 2 evolutionary phase diagram.

At present, the performance expenses incurred by the Chinese general contractor surpass twice the magnitude of the damage to its reputation. In case the benefits that the host government receives by continuing the contract through negotiations are inferior to the benefits of termination, or the benefits that the Chinese general contractor obtains by accepting the negotiation route to continue the contract are smaller than the gains of compensation obtained by suing the host government, under such circumstances, the equilibrium of the game will lead to the discontinuation of cooperation by both the host government and the Chinese general contractor. The stability of the system’s evolution is exhibited in Table 6, whereas the path of evolution is presented in Fig 2.

**Table 6:** Stability analysis of equilibrium points of Situation 2 evolutionary game system.

	$r_{1}>0,r_{2}<0$			$r_{1}<\;0,r_{2}>\;0$			$r_{1}<\;0,r_{2}<0$		
Equilibrium points	det.J	trJ	Stability	det.J	trJ	Stability	det.J	trJ	Stability
(0,0)	+	–	ESS	+	–	ESS	+	–	ESS
(0,1)	+	+	Instability point	–	+	Saddle point	–	±	Saddle point
(1,0)	–	±	Saddle point	+	+	Instability point	–	±	Saddle point
(1,1)	–	±	Saddle point	–	±	Saddle point	+	+	Instability point
$\left(x^{*},y^{*}\right)$	Beyond the boundary			Beyond the boundary			Beyond the boundary		

### Situation 3:$c_{e}-2\theta <0,r_{1}>0,r_{2}>0$

**Fig 3:**
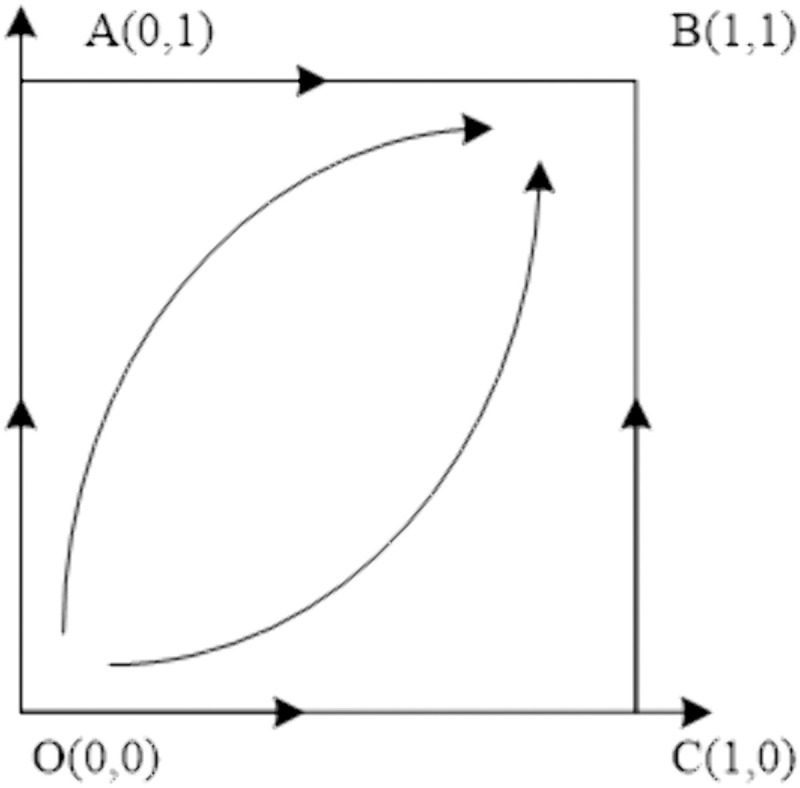
Situation 3 evolutionary phase diagram.

At present, the expense incurred by the Chinese general contractor in terms of performance is less than twice the cost of damage to its reputation. Furthermore, the host government stands to gain more from continuing the contract through negotiations than it would from terminating it, whereas the Chinese general contractor would also benefit more from accepting the bargaining process to continue the contract than it would from seeking compensation via legal action against the host government. Given this situation, the equilibrium point of the game is reached when both the host government and the Chinese general contractor continue to cooperate with one another. The stability of the system is demonstrated in Table 7, and the evolutionary path is illustrated in Fig 3.

**Table 7:** Stability analysis of equilibrium points of Situation 3 evolutionary game system.

Equilibrium points	det.J	trJ	Stability
(0,0)	–	±	Saddle point
(0,1)	–	±	Saddle point
(1,0)	+	+	Instability point
(1,1)	+	–	ESS
$\left(x^{*},y^{*}\right)$	Beyond the boundary		

### Situation 4:$c_{e}-2\theta <0,r_{l}>0,r_{2}<0$

**Fig 4:**
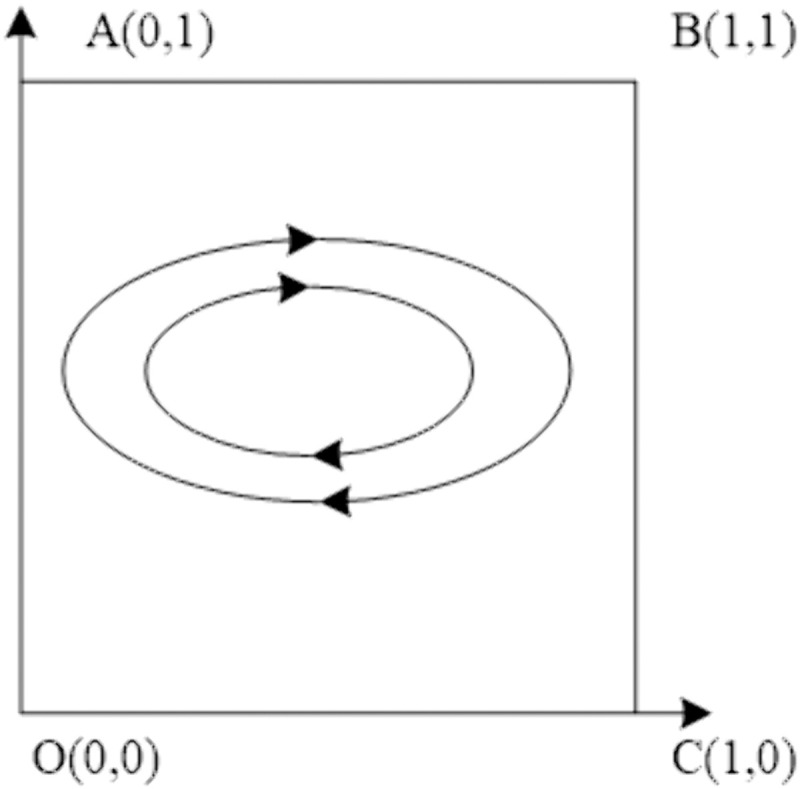
Situation 4 evolutionary phase diagram.

At the present juncture, the performance expenses incurred by the Chinese general contractor are inferior to twice the reduction in reputation. In addition, the benefits reaped by the host government through continued negotiation of the contract outweigh the advantages of contract termination. Conversely, the benefits acquired by the Chinese general contractor through continued negotiations with regards to the continuation of the contract are inferior to the compensation gained from suing the host government. Given these circumstances, the final system assumes a central position. The evolutionary stability of this system is depicted in Table 8, whereas the evolutionary path is illustrated in Fig 4.

**Table 8:** Stability analysis of equilibrium points of Situation 4 evolutionary game system.

Equilibrium points	det.J	trJ	Stability
(0,0)	–	±	Saddle point
(0,1)	–	±	Saddle point
(1,0)	–	±	Saddle point
(1,1)	–	±	Saddle point
$\left(x^{*},y^{*}\right)$	+	0	Center Point

### Situation 5:$\;C_{e}-2\theta <0,\;r_{1}<0\;,r_{2}>0\;or\;r_{2}<0$

**Fig 5:**
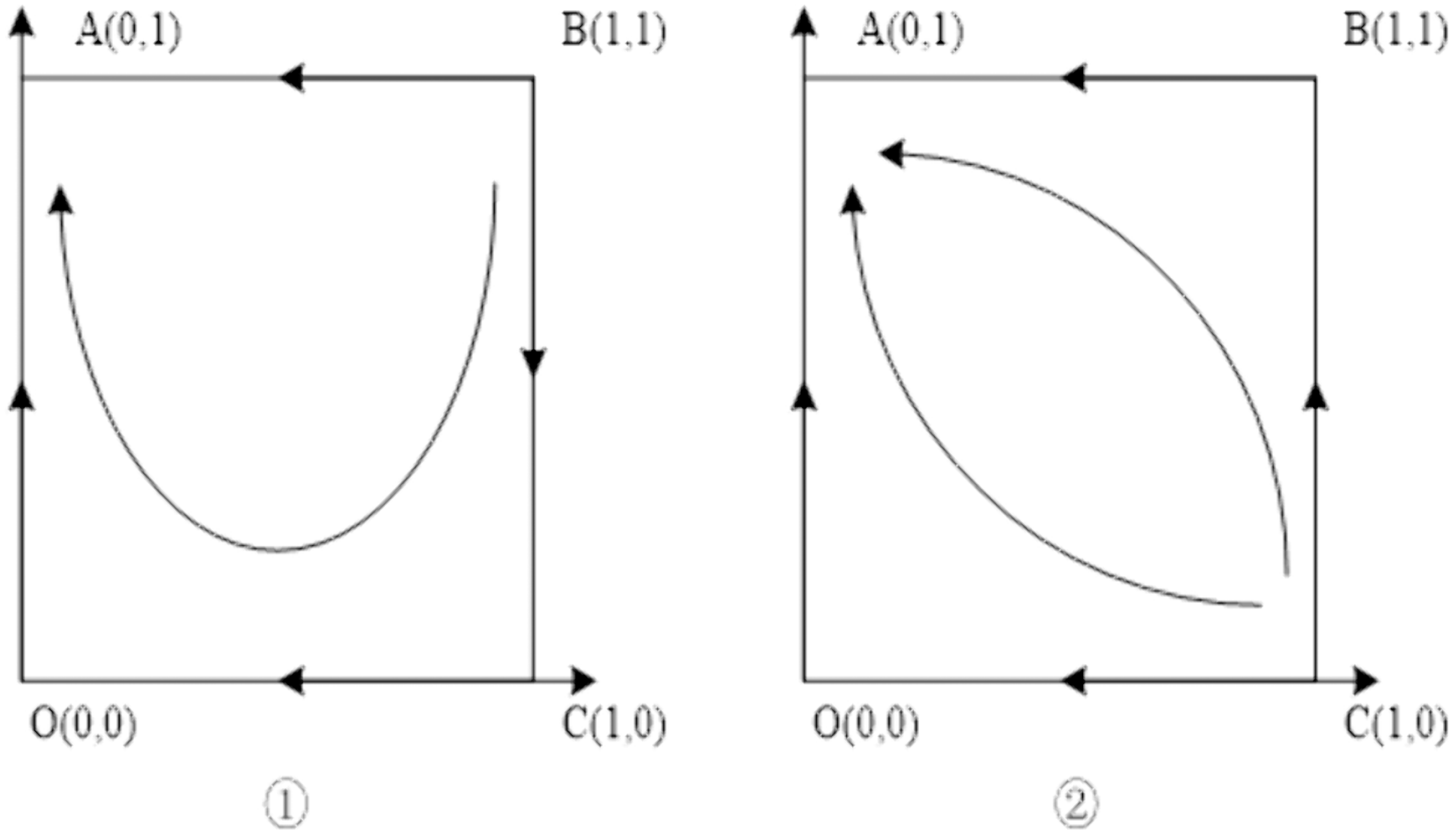
Situation 5 evolutionary phase diagram.

At this point, the cost of performance incurred by the Chinese general contractor is below twice the expense of reputational loss, and the benefits derived from negotiations for continuing the contract with the host government are inferior to those gained from terminating the contract. Irrespective of whether the advantages of the Chinese general contractor accepting the method of negotiation for continuing the contract surpass the benefits of compensation acquired by litigating against the host government, the game equilibrium will ultimately converge towards the discontinuation of cooperation by the host government and the continuation of cooperation by the Chinese general contractor. The stability of the system’s evolution may be observed through Table 9, whereas the evolution path may be traced through Fig 5.

**Table 9:** Stability analysis of equilibrium points of Situation 4 evolutionary game system.

	$r_{2}>0$			$r_{2}<0$		
Equilibrium points	det.J	trJ	Stability	det.J	trJ	Stability
(0,0)	–	±	Saddle point	–	±	Saddle point
(0,1)	+	–	ESS	+	–	ESS
(1,0)	+	+	Instability point	–	±	Saddle point
(1,1)	–	±	Saddle point	+	+	Instability point
$\left(x^{*},y^{*}\right)$	Beyond the boundary			Beyond the boundary		

## 5. Matlab numerical simulation analysis of key bargaining factors

The pertinent parameters are chosen for sensitivity analysis using the Matlab software, and the parameter values are subsequently altered to investigate the impact of these parameters on the stable strategies of the game equilibrium for both parties of the game. An array of cases is explored, solved, and scrutinized to analyze the position and stability of the equilibrium point of the evolutionary game. The system’s evolutionary process is depicted by a phase diagram. The values of the parameters will affect the speed of convergence of the system to the equilibrium point in the evolutionary process, set the initial strategy x=0.6, y=0.3, and other parameters are taken as shown in Table 10.

**Table 10:** Parameter values table.

Parameters	$\pi_{g}$	$\pi_{e}$	$c_{g}$	$c_{e}$	l	θ	μ	p	q	d
**Values/Unit**	8	10	1	2	3	4	6	5	2	3

### 5.1 The impact of Chinese general Contractor negotiation cost on the Evolutionary Path

**Fig 6:**
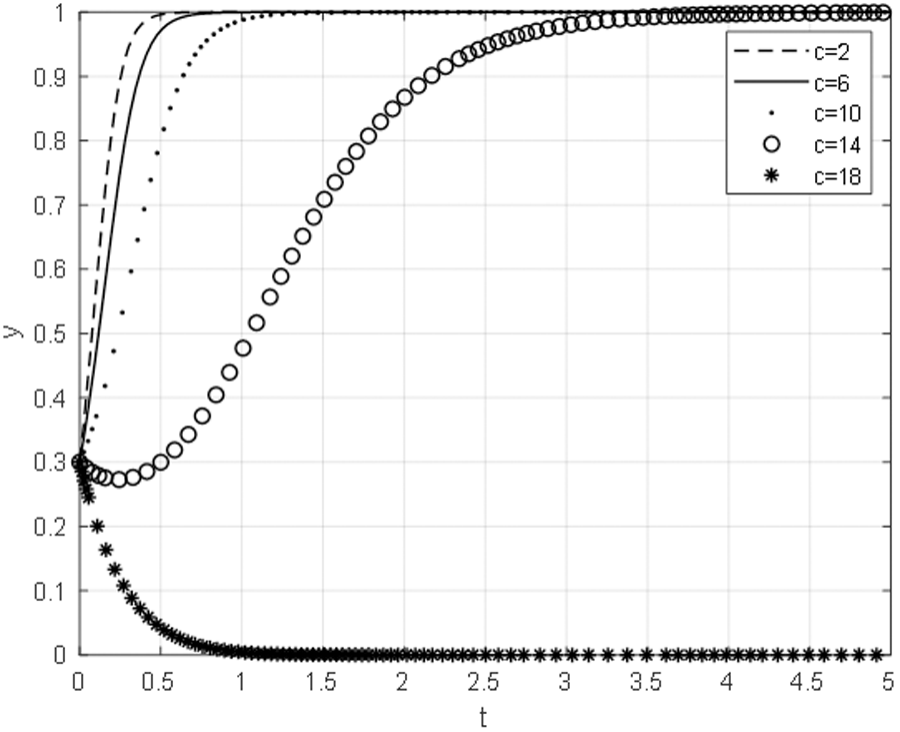
The impact of negotiation cost on the evolutionary path of Chinese general contractor.

With other parameters unchanged, the negotiation cost $c_{e}$of the Chinese general contractor is taken as 2,6,10,14,18, and the evolutionary path of the equilibrium strategy under different negotiation cost is obtained, as shown in Fig 6. As the cost of negotiation for the Chinese general contractor diminishes, the strategic approach of the aforementioned contractor gravitates towards the direction of continued collaboration, as opposed to terminated collaboration. Furthermore, it can be inferred that the lower the negotiation cost incurred by the Chinese general contractor, the more expeditious the contractor’s convergence toward the direction of continuation.

When the cost of negotiation for the Chinese general contractor is low, the optimal strategy for the aforementioned contractor during bargaining negotiations is to persist in their cooperation. The optimization of the organizational structure of negotiations, coupled with the reduction of negotiation time, not only decreases the cost of negotiation for the Chinese general contractor but also minimizes the losses incurred by project suspension. Additionally, it increases the likelihood of the Chinese general contractor choosing to continue with the construction project. The Chinese concepts of sharing, co-construction, joint discussion, and coordination, as opposed to Western litigation or contract management, are effective in precisely shortening the negotiation time.

### 5.2 The impact of host government negotiation cost on the evolutionary path

**Fig 7:**
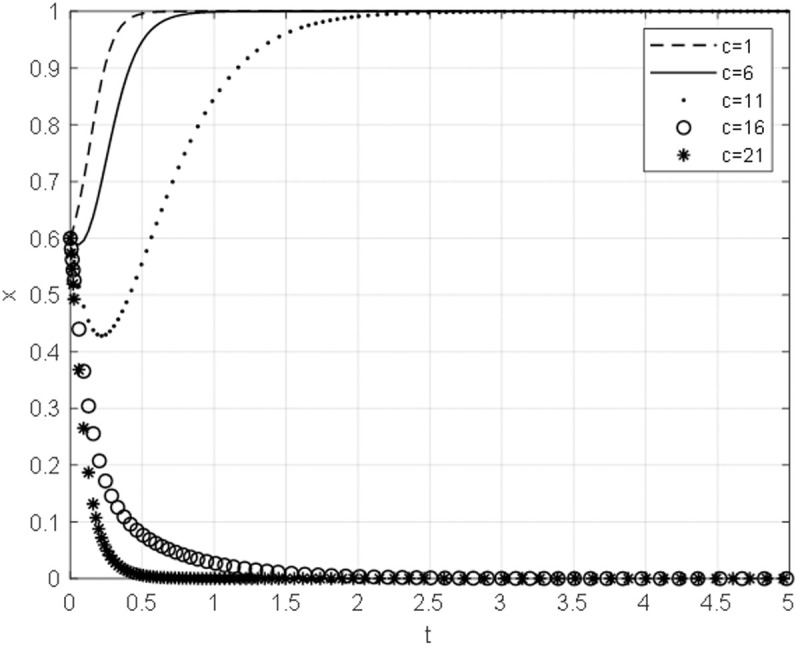
The impact of negotiation cost on the evolutionary path of the host government.

Taking the negotiation cost $c_{g}$ of the host government as 1,6,11,16,21 with other parameters unchanged, the evolution path of the equilibrium strategy under different negotiation cost is obtained, as shown in Fig 7. As the expenses associated with negotiation incurred by the host government diminish, the host government aligns its strategy towards a shift from terminated cooperation to ongoing cooperation. Moreover, it is observed that the greater the reduction in the host government’s negotiation expenditure, the more swiftly the host government converges towards the path of sustained cooperation.

When the cost of negotiation for the host government is low, the most advantageous course of action for the said government during bargaining negotiations is to continue cooperation. This can be achieved by minimizing the number of negotiations and clearly outlining the demands of the negotiation. In doing so, the negotiation cost for the host government is reduced. Furthermore, a decrease in negotiation time leads to a decrease in compensation paid to the Chinese general contractor by the host government. This, in turn, increases the likelihood that the host government will choose to proceed with the construction project. The introduction of China’s concept of harmony and cooperation [The concept of harmony and cooperation: The concept of harmony and cooperation is the primary value of Chinese culture, the essence of Chinese culture, and the most perfect and complete form of embodiment of Chinese cultural life.] differential order pattern [Differential order pattern: “Differential order pattern” is proposed by Fei Xiaotong, which occurs in social relations such as kinship and geopolitical relations, and takes oneself as the center and pushes away like water ripples, further and further, thinner and thinner and can be released and retracted, and it produces different circles with the change of time and space in which one is located.], relationship governance [Relationship governance: Relationship governance refers to the management approach of controlling enterprises formed by balancing various relationships through human governance, which has Chinese characteristics and is a traditional corporate governance method developed from state-run enterprises.] and order governance [Order Governance: Chinese indigenous ritual governance is a concept of order governance represented by Confucianism and widely recognized in the integration of traditional culture and governance theory to regulate social behavior.] into risk management can precisely reduce negotiation cost.

### 5.3 The impact of the bargaining amount on the evolutionary path of both parties

**Fig 8:**
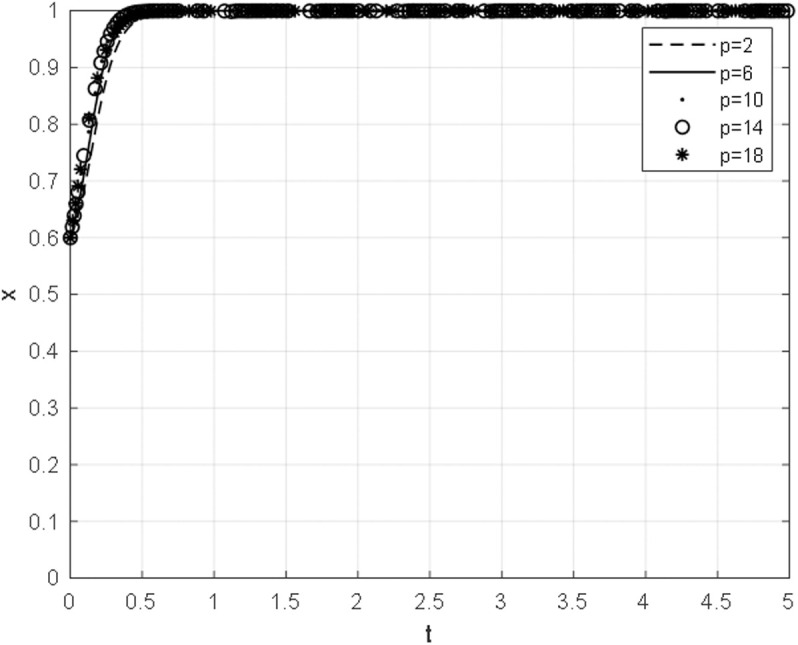
The impact of bargaining amount on the evolutionary path of the host government.

**Fig 9:**
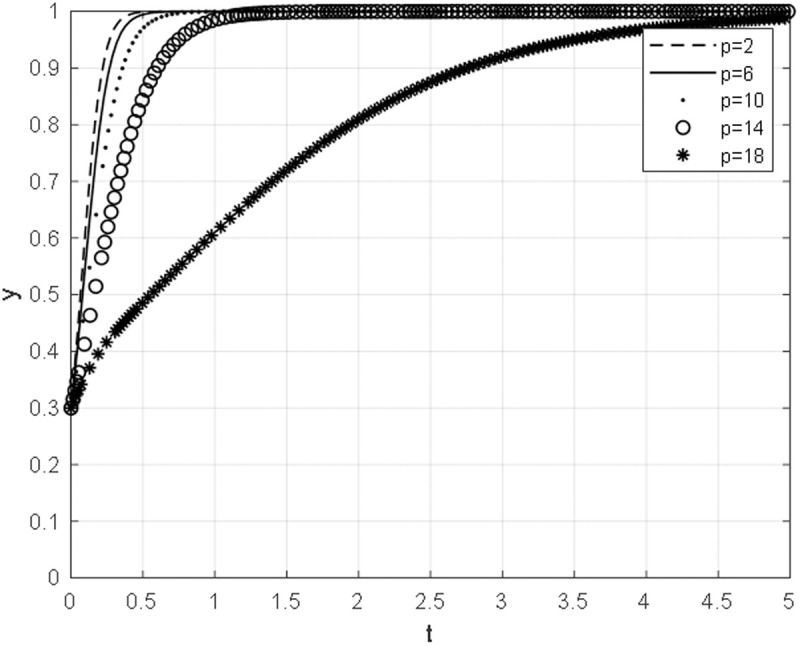
The impact of bargaining amount on the evolutionary path of the Chinese general contractor.

The evolutionary paths of equilibrium strategies under different bargaining amounts are obtained by taking the bargaining amount p as 2,6,10,14,18 with other parameters unchanged, as shown in Figs 8 and 9. As the amount of bargaining by the host government increases, the host government’s strategy converges to the direction of continued cooperation at a faster rate, while the convergence of the strategy of the Chinese general contractor toward continued cooperation slows down. Furthermore, the figure reveals that the impact of the bargaining amount on the convergence speed of the Chinese general contractor’s strategy is more pronounced than that on the convergence speed of the strategy of the host government.

In the process of bargaining negotiation, as the bargaining amount p continues to increase, $r_{1}>0$ is always established. When p increases to $p>\pi_{e}-d+2l+2\theta -c_{e}$, that is, $r_{2}$ changes from positive value to negative value, the dominant strategy of both parties in the negotiation changes from continued cooperation to an unstable state, and the strategy selection of both parties cannot converge. The alteration in circumstances corresponds to a modification in the advantages bestowed upon the Chinese principal contractor. This elucidates the fact that the amount of bargaining influences the Chinese principal contractor’s strategy convergence rate more than the host government’s strategy convergence rate. Consequently, during the bargaining negotiation, the host government ought to explicate the current difficulties instead of initiating “unwarranted” high offers that would be detrimental to the Chinese principal contractor and solely serve its own interests. China promotes the notion of harmony, order, and adaptability, which can balance the interests of all parties.

## 6. Discussion

Through the mathematical derivation and simulation analysis above we find that continued cooperation is not easy to achieve, and the evolutionary stabilization strategies of both parties are related to several key parameters, namely the difference between the benefits of continuing and terminating the contract for the host government and China, respectively, and the difference between the cost of performance and the loss of reputation of the Chinese contractor by a factor of two. It can be seen that the host government only needs to consider the pure benefits of cooperation or termination, while the Chinese contractors need to consider the additional costs.

Only two of the previous five scenarios achieve an evolutionarily stable strategy in which both parties continue to cooperate. The first thing that is certain is that both parties are likely to continue to cooperate only if the benefits of continuing to do so are greater than the benefits of terminating the cooperation. The difference lies in the trade-off between the cost of performance by the Chinese contractor and the loss of its reputation. When the Chinese contractor is unwilling to bear a high loss of reputation or faces a low cost of performance, continuing the cooperation is a result that both parties would like to see; however, when the loss of reputation is not so terrible or the cost of performance is too high, the situation becomes unpredictable, and whether the two parties will continue the cooperation or terminate depends on the probability of the two parties’ strategic choices. When the benefits of continuing the contract for either party are negative, it is difficult to achieve a state in which both parties continue to cooperate. When both parties have negative benefits of continuing cooperation and the performance cost of the Chinese contractor is very large, the termination of cooperation is indisputable; when the loss of reputation of the Chinese contractor is even greater, the Chinese contractor is always inclined to make up for the contract in order to continue to cooperate with the host government, but its own loss of continuing cooperation or the host country’s denial of the continued cooperation in the face of the loss of the role of the impediment.

## 7. Implications

The discussion in the previous chapter shows that sustained cooperation between the host government and the contractor occurs only under two conditions. Accordingly, the governance of bargaining risk in Belt and Road infrastructure projects needs to be assessed in terms of mutual benefits, performance costs, and reputational loss. At the same time, recent research indicates that introducing process-oriented principles—anticipation, inclusion, reflexivity, and responsiveness—can shift negotiation from adversarial interaction to co-governance, thereby reducing bargaining costs and stabilizing expectations in cross-border projects [31]. This mechanism-level insight is highly consistent with our Eastern governance approach. Against this backdrop, this chapter introduces related theories and concepts, including Eastern governance, and, together with illustrative cases, seeks to contribute to bargaining-risk governance in infrastructure projects beyond the context of the Belt and Road Initiative.

### 7.1 Business and management implications: from the governance of bargaining for the “Belt and Road” infrastructure projects

#### (1) The concept of harmony and cooperation.

As the quintessence of traditional culture, the “spirit of harmony and cooperation” is considered the “meta-spirit” of China’s autochthonous governance theory. In contrast to Western theories that prioritize rational thinking, the notion of harmony and cooperation accentuates the amalgamation of reason and emotion in managing social relationships. The culture of “harmony and cooperation” is explicated as “the sum of the conflict and fusion of many elements of nature, society, interpersonal relationships, the human mind and civilization, and the new life and new things formed by the recombination of elements in the dynamic process of conflict and integration [32].” The Chinese people have consistently pursued harmonious development, win-win cooperation, and collaboration among all nations. Over the course of its extensive historical development, this concept of harmonious coexistence and differences has become deeply ingrained in the national culture and character of the Chinese people, serving as the fundamental principle for China’s approach to international relations and social issues [33]. In response to the bargaining risk of the “Belt and Road” infrastructure projects, China consistently upholds the concept of harmony and cooperation, endeavoring to govern the bargaining risk by employing the path of integration and integrative thinking as the main method.

The China–Laos Railway Project, a landmark infrastructure initiative under the Belt and Road Initiative, offers a compelling example of how the concept of harmony and cooperation—as the meta-spirit of China’s indigenous governance philosophy—can be translated into practical risk mitigation in cross-border bargaining contexts. During the early stages of the project, differences in legal systems, construction standards, and financial responsibility generated negotiation deadlocks and elevated transaction costs for both sides. While a Western-style adversarial negotiation might have pushed the parties toward contract litigation or renegotiation, the Chinese side instead embraced a path guided by integrative thinking rooted in the logic of harmony.

Rather than viewing conflict as zero-sum, Chinese negotiators treated the disputes as part of a dynamic integration process—seeking not compromise through subtraction, but coexistence through mutual adaptation. For instance, a trilateral mechanism was established involving Chinese engineers, Lao transport authorities, and third-party regional coordinators to harmonize technical standards and training protocols. Additionally, cultural engagement initiatives—such as language training, joint holiday celebrations, and symbolic co-governance rituals—fostered emotional resonance and trust between the parties, reducing miscommunication and bureaucratic friction.

This harmony-oriented approach effectively lowered the negotiation costs ($c_{g}$, $c_{e}$ decreased), mitigated potential reputational losses (μ, θ decreased) by reducing the risk of conflict escalation, and stabilized the bargaining environment by avoiding sharp fluctuations in bargaining demand (p stabilized). Ultimately, the project proceeded smoothly despite early tensions, demonstrating how harmony and cooperation—unlike rigid legalism—can function as a flexible yet robust governance tool in the face of complex bargaining dynamics. The China–Laos Railway thus exemplifies how a deeply rooted cultural principle can evolve into a strategic governance mechanism, particularly suited for infrastructure diplomacy under the BRI framework.

#### (2) The concept of sharing economy.

In contemporary times, the efficacy of economic tenets such as the international division of labor and comparative advantage in guiding corporate strategies and prognosticating project implementation is diminishing, particularly in the context of the Belt and Road infrastructure projects, as reported by Durand and Kremp [34]. As such, the sharing economy concept serves as a salient complement. In contrast to the conventional concept of incessantly augmenting competition to secure advantages and efficiency, the sharing economy concept is centered on cooperation for competitive advantages and efficiency, particularly with regard to the balance of competition and cooperation concepts, as elucidated by Dyal-Chand, Ika et al., and Xue et al. [35–37]. Consistent with this logic, recent research advances a stakeholder “affordance” perspective: by purposefully designing interaction modalities—such as data sharing, co-monitoring, and co-benefit allocation—project organizations can enable cooperative, pro-sustainability behaviors and thereby lower bargaining and coordination costs in BRI negotiations [38]. The sharing economy concept has the potential to supplant existing economic theories as the dominant paradigm in the next 30 years, impacting various sectors and engendering a novel social, legal, moral, political, economic, and cultural system grounded in digitization, according to Kawashima [39]. Professor Eric has dubbed the sharing economy concept as “the second invisible hand” and posits that it will undoubtedly extend to the planning, investment, construction, operation, management, and maintenance of extant transportation, water, and energy infrastructure facilities, as posited by Piscicelli, Ludden, and Cooper, Yeow et al., Artto et al., and Patanakul and Shenhar [40–43], thereby yielding unforeseen outcomes for the governance of the bargaining risk of the “Belt and Road” infrastructure projects.

The advent and evolution of project sharing have had a significant impact on traditional project management theory and practice and have been instrumental in addressing gaps in project theory [44,45]. Infrastructure projects have garnered attention due to shared elements such as benefits, values, resources, information, risks, and ideas. However, there is a lack of a systematic governance framework [46–49]. Moreover, shared environments will become a crucial basis for measuring international project governance capabilities. Shared systems have significantly diminished the previous advantages of firms, and resource- and competition-based perspectives may even undermine and hinder project innovation practices [50–52]. Therefore, the “Belt and Road” infrastructure projects must introduce the concept of sharing to exploit the benefits of cooperation. The sharing economy theory (similar to the old sharing economy of Nobel laureate Meade and Nobel nominee Weitzman) and the extensive involvement of infrastructure project practices in the “Belt and Road” Initiative have created opportunities for achieving breakthrough results in infrastructure project governance research and practice.

The Sahiwal Coal Power Plant Project, a flagship energy infrastructure project under the China–Pakistan Economic Corridor (CPEC), offers a concrete embodiment of the sharing economy concept in action—where cooperation, rather than pure competition, becomes the vehicle for strategic advantage. Unlike traditional models of project management grounded in unilateral control and resource competition, this project integrated mechanisms for shared resources, joint responsibility, and mutual benefit, reflecting the theoretical shift toward collaborative governance in transnational infrastructure delivery.

From the early planning phase, both Chinese and Pakistani stakeholders embraced shared protocols in energy planning, environmental regulation, and risk allocation. For example, the integration of environmental protection facilities was not only funded by the Chinese contractor but jointly monitored by a binational commission. A real-time data-sharing platform was established to ensure operational transparency, while contractual arrangements stipulated dynamic electricity pricing adjusted via cooperative negotiation rather than unilateral imposition. These mechanisms reflect a transition from firm-centric ownership to ecosystem-wide co-creation, a hallmark of the second invisible hand of the economy as envisioned by Piscicelli et al. [43].

In the language of bargaining risk governance, this “shared infrastructure model helped reduce the frequency and intensity of price-related renegotiations (p stabilized), minimized procedural uncertainty ($c_{g}$, $c_{e}$ lowered), and enhanced the alignment of long-term interests ($\pi_{g}$, $\pi_{e}$ increased). More importantly, it promoted a culture of relational reciprocity, mitigating trust deficits and reputational losses (μ, θ decreased). The Sahiwal case exemplifies how sharing economy principles—applied not merely to consumer platforms but to sovereign-scale infrastructure projects—can reshape the dynamics of cooperation, turning potential conflicts into enduring partnerships.

#### (3) The concept of project management.

In the context of the sharing economy concept, there is increasing attention paid to the balance between competition and cooperation in the strategic adjustment of international enterprises [53]. The function of project management in carrying corporate strategy is weakening, leading to an increase in the independence of projects [54]. In fact, project management has become the “compass” and “trigger” of corporate strategy adjustment [55]. Therefore, it is imperative that the laws presented by this bargaining phenomenon be followed in the international market [56,57], rather than demonizing and politicizing these projects, which would only initiate bargaining behavior. Such behavior is contrary to the original intention of the “Belt and Road” Initiative and the Eastern concept. The sharing economy concept can guide the formation of a new project management model for international infrastructure [58]. However, no scholars have yet proposed a project-sharing model, nor have they introduced these concepts into the governance of the bargaining risk of the “Belt and Road” infrastructure projects and other issues. Furthermore, the causes, concepts, values, and obstacles of project sharing have yet to be analyzed in-depth, and the project management academic community is far behind the practice community in this regard.

In the context of the East Coast Rail Link (ECRL) project in Malaysia, the evolution of project management has reflected the theoretical shift from being a passive implementer of enterprise strategy to becoming an active “compass” and “trigger” of strategic adaptation [55]. The initial phase of the ECRL project saw delays and uncertainty caused by Malaysia’s political transition in 2018, during which the project was suspended, renegotiated, and finally reinstated. This fluctuation mirrored not only political dynamics but also the lack of a flexible and autonomous project governance structure.

In response, the Chinese contractor adopted a project-centered governance approach, treating the ECRL project as an independent strategic entity rather than a mere contractual deliverable. Several concrete mechanisms were introduced, including a local project joint task force, decentralized financial negotiation teams, and explicit risk-sharing clauses in renegotiated contracts. These adjustments effectively transformed project management into a strategic buffer zone insulated from political volatility—consistent with above proposition that project governance is evolving toward shared and decentralized control structures in the sharing economy context [58].

This governance transformation significantly reduced the Chinese contractor’s exposure to potential market loss (l decreased), minimized credibility damage (θ decreased), and lowered negotiation costs ($c_{e}$ lowered) through more localized and autonomous coordination. Meanwhile, the Malaysian government was able to regain control over sensitive political narratives, reducing reputational losses (μ reduced) and eventual compensation obligations (d reduced).

The ECRL case exemplifies the practical emergence of a project-sharing governance model, even in the absence of a formalized academic framework. It underscores the urgency of aligning academic research on project management with practical innovations in global infrastructure governance under the Belt and Road Initiative. As your original manuscript emphasizes, demonizing or politicizing international projects tends to incite bargaining behavior rather than fostering cooperative adaptation. Project-level autonomy and decentralization offer a more resilient model, both theoretically and operationally, for managing bargaining risk in transnational contexts.

### 7.2 Theoretical implications: from the governance of bargaining for the “Belt and Road” infrastructure projects

#### (1) The theory of differential order pattern.

The theory of Eastern governance has been passed down for millennia, and although it may not be mainstream in the microcosm of corporate and project governance, its significance in social governance and other areas cannot be disregarded. Fei Xiaotong’s book Rural China introduced the notion of a “differential order pattern” to describe the social structure and interpersonal relationship characteristics of traditional Chinese society. Fei Xiaotong compares the “group pattern” of Western society with the “differential order pattern” of Chinese society in order to explain the latter. The relationship between people in the group pattern is described as “a bundle of clearly tied firewood”, where the interpersonal relationship is succinct and equal. Chinese rural society’s interpersonal pattern of closeness and distance, on the other hand, is akin to a ripple on the water surface [59]. It extends from oneself, circle by circle, dividing closeness and distance according to the distance from oneself. This mode of governance, which relies on differential order pattern theory, has sustained the relationship between the countryside and the village for thousands of years.

In the context of the Kyaukpyu Deep-Sea Port Project in Myanmar, initial project implementation was obstructed by widespread local resistance, particularly from coastal fishing communities, due to perceptions of unequal land compensation and limited local benefit-sharing. Traditional contractual governance models, which emphasize clear boundaries and standardized procedures, proved inadequate in addressing such localized, trust-based concerns.

To respond to this, the Chinese side gradually adopted a relational governance model aligned with Fei Xiaotong’s theory of “differential order pattern.” Rather than interacting uniformly with all stakeholders, Chinese contractors prioritized engagement based on concentric circles of social distance—starting with local government officials, then village elders, and eventually informal community leaders. For example, the project team initiated temple restoration programs and fishing equipment subsidies for influential coastal households, which catalyzed broader community acceptance. These targeted efforts mirrored Fei’s conceptualization of relationships as “ripples on a water surface,” extending from the self outward in layers of closeness and moral obligation [59].

Practically, this governance adaptation reduced local hostility and bargaining intensity (reflected in a lower p), improved host acceptance (raising the effective cooperation benefit $\pi_{g}$), and reduced the credibility loss from political backlash (μ decreased). The Kyaukpyu case illustrates how traditional Eastern governance concepts—though historically rooted in rural societies—can offer effective, context-sensitive mechanisms for managing bargaining risks in international infrastructure projects.

#### (2) The theory of relationship governance.

Rational governance frequently presents itself as a counterpoint to rule-based governance, indicating that the governance process is not strictly rule-based, but rather consensual, reliant upon mutual trust and full consultation among various participants. The roots of relational governance are grounded in the Eastern governance system, where cultural practices hold significant influence. Chinese society, for instance, has been an “acquaintance society” since ancient times, while Western governance culture is based on the rule of law and regulations as the foundation of governance. While rule-based governance is essential in governance activities, relational governance can serve as an alternative and complement to rule-based governance in certain contexts. In the realm of project governance, relational governance can largely compensate for the absence of “contractual governance” in projects, and has emerged as one of the two most vital concepts or approaches in corporate governance and project governance.

The Jakarta–Bandung High-Speed Railway Project in Indonesia exemplifies the growing prominence of relational governance as an alternative to strict rule-based or contract-based governance, particularly in cross-border infrastructure cooperation. In the early stages of the project, disputes over land acquisition, unclear property rights, and inconsistent policy enforcement led to significant delays and stakeholder mistrust. These challenges revealed the limitations of formal contracts in environments where the legal system is fragmented and administrative power is decentralized.

To address these issues, the Chinese project consortium did not rely solely on legal renegotiation mechanisms. Instead, they adopted a relational governance approach deeply rooted in Eastern governance traditions—emphasizing mutual trust, long-term relationships, and frequent consultation. Practical measures included establishing an Indonesian–Chinese coordination office embedded within the local government structure, frequent informal dialogues with provincial authorities, and public forums with affected communities. In one notable example, Chinese and Indonesian executives jointly attended Ramadan events in affected areas, strengthening interpersonal bonds and demonstrating mutual respect.

This relational approach significantly reduced negotiation costs ($c_{e}$, $c_{g}$), as consensus-building supplanted adversarial legal processes. It also mitigated reputational losses stemming from early-stage conflict (μ, θ), and partially offset potential market losses for the contractor by restoring local legitimacy (l stabilized). The Jakarta–Bandung case illustrates how relational governance, grounded in trust and cultural affinity, can serve as a flexible yet effective complement to rule-based governance in managing bargaining risks under the Belt and Road Initiative.

#### (3) The theory of order governance.

The traditional Chinese ritual order is composed of two orders: “constructive order” and “self-generated order”. This enables citizens of society to comply with the obligatory rules of the state, while simultaneously instilling moral standards that promote self-discipline and discipline. As a result, it serves as an effective mechanism of governance. The Chinese indigenous ritual governance, represented by Confucianism, is a theory of order and governance that is widely recognized as a fusion of traditional culture and governance theory for regulating social behavior [60]. Unlike the Western theory of social order governance, which is based on contract theory, the indigenous Chinese theory of social governance emphasizes the rule of law in conjunction with the rule of morality. From ancient times to the present, social order has been well ordered under this theory of governance, whether it was during the feudal dynasties or in modernized China. This is sufficient to demonstrate the validity of this theory in specific scenarios.

The Colombo Port City Project in Sri Lanka, a flagship Belt and Road Initiative project, encountered substantial regulatory uncertainty during its early stages, including abrupt policy reversals, environmental protests, and inconsistent licensing procedures. These challenges were symptomatic of fragmented legal enforcement and a lack of shared normative frameworks between project stakeholders. Initial attempts to rely solely on contract-based dispute mechanisms failed to resolve recurring governance frictions.

To address this, the Chinese consortium implemented a dual-layered governance mechanism reflective of traditional Chinese order governance theory. Specifically, a “constructive order” was established through the signing of memoranda of understanding (MOUs) with multiple levels of the Sri Lankan government, ensuring formal alignment with national development goals and legal standards. Concurrently, a “self-generated order” was cultivated through non-contractual moral commitments, including jointly issued ethical codes, environmental charters, and publicly endorsed social responsibility pledges signed by both parties.

These efforts promoted a holistic behavioral discipline across the project ecosystem. They not only reduced transaction friction but also fostered moral reciprocity and institutional legitimacy. As a result, reputational risks on both sides diminished (μ, θ), the host government’s project completion losses were mitigated (q decreased), and negotiation costs ($c_{e}$, $c_{g}$) fell due to fewer procedural disruptions. The Colombo case demonstrates how Confucian-rooted order governance—combining formal legal instruments with culturally resonant moral norms—can effectively stabilize cooperation in complex international infrastructure projects where rule-based governance alone may fall short.

## 8. Conclusions

This study focuses on the bargaining behavior observed in “Belt and Road” infrastructure projects, and puts forward the concept of bargaining risk as a distinct and practical form of project risk. Unlike traditional political risk, which often stems from sudden, top-down government intervention, bargaining risk typically arises within the existing contractual and institutional framework through deliberate, strategic interactions. Clarifying this distinction helps refine our theoretical understanding of international infrastructure risks and offers a more precise lens for analyzing China’s overseas projects.

Drawing on the logic of the Kindleberger Trap Theory, the research interprets these bargaining behaviors as structural manifestations of responsibility asymmetry in a rising-power context. Much like how the Kindleberger Trap describes global instability caused by hegemonic vacuums, we observe that when Chinese contractors assume de facto infrastructure leadership roles without corresponding normative legitimacy or reciprocal obligations from host states, it creates bargaining incentives. This perspective helps explain why host countries may continue to seek new terms, even within existing agreements, and why the political burden of stabilization often falls disproportionately on the Chinese side.

Through multiple case studies, the research identifies factors that commonly trigger bargaining behavior—such as political transitions, contract asymmetries, or localized resistance. Projects like the Colombo Port City, Kyaukpyu Deep-Water Port, and Jakarta–Bandung High-Speed Railway provide concrete illustrations of how tensions evolve and how governance responses vary in effectiveness.

An evolutionary game model is employed to simulate the strategic decisions of both parties. The results indicate that low negotiation costs and moderate bargaining amounts foster continued cooperation, while excessive bargaining demands, particularly when contractor gains fall below legal or reputational losses, lead rationally to project termination. These insights offer a useful framework for anticipating and managing risk in real-world infrastructure negotiations.

In addition to quantitative modeling, this study introduces three Eastern governance perspectives—differential order pattern, relational governance, and order governance—as tools for understanding and managing bargaining risks. Rooted in Chinese governance traditions, these frameworks emphasize trust, moral order, and relational equilibrium, offering practical pathways to reduce friction, stabilize expectations, and build informal legitimacy. By integrating them with the Kindleberger Trap perspective, we gain a more holistic understanding of both structural tensions and local governance solutions.

While the current analysis focuses on bilateral bargaining interactions, future research may explore multilateral negotiation dynamics, especially in projects involving international lenders, third-party contractors, or transnational regulations. Moreover, as BRI initiatives move into emerging domains such as green infrastructure, digital connectivity, and climate resilience, it becomes increasingly important to test the applicability and boundaries of this model across sectors. Further studies in these directions may extend both the theoretical relevance and policy utility of the bargaining risk framework.
